# Cancer: tilting at windmills?

**DOI:** 10.1186/1476-4598-12-108

**Published:** 2013-09-24

**Authors:** Prakash Kulkarni, Takumi Shiraishi, Rahul V Kulkarni

**Affiliations:** 1 Department of Urology, The Johns Hopkins University School of Medicine, 600 N Wolfe St, 105B Marburg, 21287, Baltimore, MD, USA; 2 Department of Oncology, James Buchanan Brady Urological Institute, Johns Hopkins University School of Medicine, Baltimore, MD, USA; 3 Department of Physics, University of Massachusetts, Boston, MA, USA

## Abstract

One of the striking characteristics of cancer cells is their phenotypic diversity and ability to switch phenotypes in response to environmental fluctuations. Such phenotypic changes (e.g. from drug-sensitive to drug-resistant), which are critical for survival and proliferation, are widely believed to arise due to mutations in the cancer cell’s genome. However, there is growing concern that such a deterministic view is not entirely consistent with multiple lines of evidence which indicate that cancer can arise in the absence of mutations and can even be reversed to normalcy despite the mutations. In this Commentary, we wish to present an alternate view that highlights how stochasticity in protein interaction networks (PINs) may play a key role in cancer initiation and progression. We highlight the potential role of intrinsically disordered proteins (IDPs) and submit that targeting IDPs can lead to new insights and treatment protocols for cancer.

## 

In light of the incessant deluge of data that often show meager correlations to cancer cause, diagnosis or prognosis, one wonders whether cancer is indeed a genetic disease and whether all cancers arise as a result of changes in the cancer cell’s genome as is commonly believed [1,2]. Such a view implying that cancer is deterministic, perhaps raises more difficulties rather than provide a clearer understanding of the disease [3,4]. In the following, we focus on a single example which is illustrative of the kind of difficulties we are dealing with.

Conventional wisdom suggests that cancer cells contain so many mutations that their reversal to normalcy is unlikely. This belief has led to the development of treatments aimed at killing cancer cells – an ambitious aim that has been difficult, if not impossible, to achieve for most cancers. However, in 2004, a landmark paper by Felsher and coworkers [5] startled the cancer world by demonstrating that indeed, rogue tumor cells can be reformed. By conditionally turning on the oncogene c-Myc in mice hepatocytes with the Tet system, the authors induced hepatocellular carcinoma in these transgenic animals. They then turned off Myc expression in these animals and surprisingly observed that Myc inactivation resulted *en masse* in tumour cells differentiating into hepatocytes and biliary cells forming bile duct structures. This was accompanied by a rapid loss of expression of the tumour marker α-fetoprotein and increase in expression of liver cell markers such as cytokeratin 8 and carcinoembryonic antigen demonstrating how oncogene inactivation may reverse tumorigenesis in the most clinically difficult cancers. In other words, the cancerous hepatocytes had turned normal albeit, dormant. However, turning Myc on again resulted in the dormant cells redeveloping cancer. Remarkably, the authors confirmed that the genomic alterations that had occurred in the cancer cells overproducing Myc remained unchanged when the Myc-expressing cells cycled between the cancerous and ‘normal’ states!

As tantalizing as these observations were, they naturally lead to the question: how does the cancer cell turn ‘normal’ if mutations were driving tumorigenesis? In light of these, and numerous other similar observations [6-9] over the last 50 years, several groups have wondered whether genetic alterations are merely a result of cancer rather than the cause. In this brief Commentary, we would like to present an alternate view, emphasizing a stochastic rather than deterministic underpinning.

It is now widely accepted that stochasticity or noise in gene expression can give rise to phenotypic variations among clonal cells in homogeneous environments [10,11]. Thus, in response to the same stimulus, two genetically identical cells can display very different phenotypes, and it has been argued that this inherent stochasticity can serve as a key driving force for tumorigenesis [12]. However, in addition to noise in gene expression, emerging evidence indicates that the information transduced in cellular signaling pathways is also significantly affected by noise [13]. It has been proposed that, noise in these pathways may be generated by the interconnected and promiscuous nature of protein interactions that are necessary to transduce signals [13]. However, how this noise arises and what consequences it has on cell fate is poorly understood.

A potential clue [14] can be obtained from a remarkable study by Vavouri *et al*. [15] that demonstrated a strong correlation between the overexpression of intrinsically disordered proteins (IDPs) and altered physiological states in model organisms. IDPs are proteins that lack a rigid 3D structure at least *in vitro*[16]. However, a remarkable feature of most IDPs is their ability to undergo disorder-to-order transitions upon binding to their biological target (coupled folding and binding) [17]. Structural flexibility and plasticity are believed to represent a major functional advantage for the IDPs enabling them to interact with a broad range of binding partners [18]. Therefore, it was postulated that IDPs are prone to initiate promiscuous molecular interactions when overexpressed resulting in altered physiological states [15,19]. Consistent with this argument, several oncogenes [20], and other cancer-associated genes [21] that are overexpressed in cancer, encode IDPs. This idea that IDP-initiated promiscuity can result in pathological effects in the *absence* of any genetic alterations may also help explain the remarkable results of Shachaf *et al.*[5] that focus on Myc which is an IDP [20].

We hypothesized [14] that noise in protein interaction networks (PINs) contributed by the conformational dynamics of IDPs may play a critical role in phenotypic switching. More specifically, we posited that this conformational noise due to the stochastic interactions initiated by the IDPs in response to a specific input, allow the system to sample through the network interaction space and drive transitions that generate phenotypic heterogeneity. Thus, IDPs can rewire PINs and, by exploring the network interaction space, activate previously masked options potentially resulting in a transition from one state (phenotype) to another.

In this stochastic model, each cell has equal probability to undergo a specific phenotypic transition in response to the given input. Indeed, such stochasticity in phenotypic switching is also thought to underlie cellular differentiation [22], generation of induced pluripotent stem cells (iPS cells) [23] and emergence of cancer stem cells from non-stem cancer cells [24]. Implicit in our model, the PIN configuration contains information that specifies the cell’s phenotype. Furthermore, as depicted in Figure 1, we posit that the network is flexible in responding to physiological changes but robust in response to adverse perturbations. However, as the perturbation increases, there is an increasing likelihood that the PIN rewires itself by unmasking latent network connections and causes the cell to transition from one phenotype to the other; for example, from normal (non-dividing) to a malignant (proliferation) phenotype when Myc is overexpressed (Figure 1). But it is important to note that, depending on the network topology, lowering the perturbation (e.g. turning off Myc expression) can result in the PIN again rewiring itself to the normal (default) network configuration, thereby reversing the phenotypic switch (malignant to normal). Interestingly, a similar situation is encountered in stem cells where sustained Myc expression is critical to maintain the pluripotent state; downregulating Myc promotes differentiation of these cells [25]. Conversely, overexpression of Myc in dormant cells kicks them into a proliferative mode.

**Figure 1:**
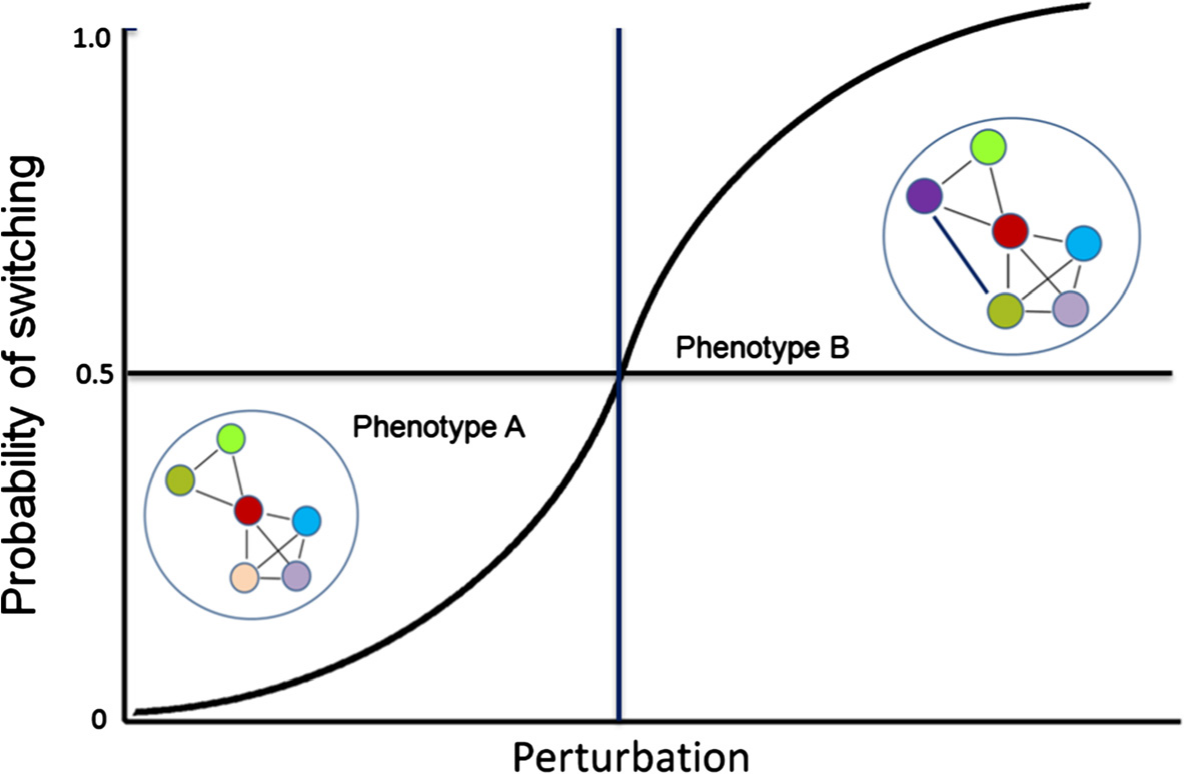
**Schematic representation of phenotypic switching driven by noise in protein interaction networks.** Phenotype A represents a normal cell that is characterized by a specific configuration of its protein interaction network (PIN). When subjected to perturbations, the levels of certain intrinsically disordered proteins (IDPs) go up and promote promiscuous interactions to rewire the PIN. If the new configurations of the rewired PIN remain within the threshold characteristic of phenotype A, this phenotype is retained notwithstanding minor fluctuations in the network topology. However, if the search unmasks latent PIN configurations that cross the threshold, the cell transitions to a cancer cell represented by phenotype B. Each cell has the same probability of switching to phenotype B, and once the perturbation exceeds a threshold (vertical line) the majority of cells in the population will be in phenotype B.

But how might information residing in PINs be transmitted so that it can be stably inherited? It is now widely accepted that information that is transmitted transgenerationally can be encoded epigenetically. Interestingly, several proteins that are involved in epigenetically sculpting the chromatin are IDPs suggesting that rewiring of protein networks could result in heritable epigenetic changes [14] and cfs therein]. Thus we conjectured [14] that, working together, these changes in the PIN instituted by the IDPs could account for stochastic phenotypic switching.

Cancer cells, like all other living (cells, organisms, and ecosystems) and many non-living systems in the universe (stars and galaxies), are self-organizing systems that exhibit nonlinear dynamics. However cancer cells, unlike their normal counterparts, exhibit traits typically associated with primitive, single-celled organisms such as bacteria that have an amazing adaptive tenacity [26,27]. As a result, it has been difficult to treat cancer. Thus, several groups [28], in particular, Huang and coworkers who have advanced the cancer attractor concept [4,29,30], have argued that there needs to be a paradigm shift from the prevailing view of cancer that is strongly influenced by fundamentally deterministic approaches. By applying the tools of nonlinear dynamics, network theory and stochastic modeling in combination with experiments to characterize cancer protein network connectivity and functionality we need to decipher how cancer cells self-organize to generate phenotypic heterogeneity by different mechanisms. This knowledge can potentially lead to a more fundamental understanding of cancer and to the development of more effective therapeutics. Perhaps, similar ideas may have already been deployed by Nature. For example, a recent study concerning certain plant pathogens found that they selectively deploy independently evolved virulence proteins that interact with a limited set of highly connected cellular hubs in order to take control of host cells [31]. The latter should serve as an inspiration, not just as a paradigm, to go after IDPs especially, those that are aberrantly expressed only in cancer cells such as the Cancer/Testis Antigens. Are we tilting at windmills instead?

## Abbreviations

IDPs: Intrinsically disordered proteins; PIN: Protein interaction network; iPS: Induced pluripotent stem cells.

## Competing interests

The authors declare that they have no competing interests.

## Authors’ contributions

PK conceived the idea. PK, TS and RVK wrote the manuscript. All authors read and approved the final manuscript.

## Authors’ information

PK, Assistant Professor. TS, Postdoctoral Fellow, and RVK is an Associate Professor.
